# Biosimilar underutilization alone does not foretell a broken biologics market

**DOI:** 10.1093/haschl/qxae090

**Published:** 2024-07-17

**Authors:** Fariel LaMountain, Molly T Beinfeld, William Wong, Eunice Kim, James D Chambers

**Affiliations:** Center for the Evaluation of Value and Risk in Health, Institute for Clinical Research and Health Policy Studies, Tufts Medical Center, Boston, MA 02111, United States; Center for the Evaluation of Value and Risk in Health, Institute for Clinical Research and Health Policy Studies, Tufts Medical Center, Boston, MA 02111, United States; Genentech, Inc., San Francisco, CA 94080, United States; Genentech, Inc., San Francisco, CA 94080, United States; Center for the Evaluation of Value and Risk in Health, Institute for Clinical Research and Health Policy Studies, Tufts Medical Center, Boston, MA 02111, United States

**Keywords:** biosimilars, biosimilar competition, originator, biologics, infliximab, pegfilgrastim, trastuzumab, filgrastim, payer coverage, average sales price, ASP, wholesale acquisition cost, WAC

## Abstract

Biosimilars offer the potential for cost savings and expanded access to biologic products; however, there are concerns regarding the rate of biosimilar uptake. We assessed the relationship between biosimilar and originator pricing, coverage, and market share by describing four case studies that fall into two categories: (1) sole preferred coverage strategy (ie, aim is to have originator product preferred; biosimilar(s) non-preferred), defined as steep average sales price (ASP) reductions for originator products (decline in net prices by at least 50% following the introduction of biosimilar competition by 2022) and (2) non-sole preferred coverage strategy (ie, aim is to have originator product preferred alongside biosimilar products), defined as moderate ASP reductions for originator products with (net prices did not decline by at least 50% of its pre-biosimilar competition value). We found that originators with sole preferred coverage strategies maintained formulary preference and market share relative to originators with non-sole preferred coverage strategies. Regardless of strategy, the market-weighted ASP for all four product families (originator and biosimilars) declined significantly in the years following the introduction of biosimilars, suggesting that biosimilar uptake alone may not be a complete measure of whether the biosimilar market is facilitating competition and lowering prices.

## Introduction

The Biologics Price Competition and Innovation Act (BPCIA) of 2009 created an approval pathway for biosimilar products with the goal of introducing competition, potentially driving down prices, and making therapies more affordable and accessible for patients.^1^ Biosimilars are biologic products that are “highly similar” and do not discernibly differ from the originator biologics in terms of safety or effectiveness.^2^ Some sources have estimated the potential savings from biosimilars between 2021 and 2025 to be $38.4B.^3^ However, discussions about the savings from biosimilars often assume that the increased uptake and utilization of biosimilars are primary drivers of potential savings, highlighting lack of interchangeability, manufacturer lawsuits, and intellectual property secrecy as significant barriers to adoption.^4-6^

Comparisons in biosimilar utilization with countries outside the United States may contribute to this belief. For example, the European Medicines Evaluation Agency (EMA) has approved 75 biosimilars compared to only 45 biosimilar approvals in the United States. However, the EMA approved its first biosimilar in 2006, while the United States did not approve its first biosimilar through the BPCIA-established pathway until 2015. Despite the almost 9-year head start, this disparity in the absolute number of biosimilar approvals may contribute to a belief that the US biosimilar market is suboptimal. Encouragingly, a recent study showed that biosimilar market share increased at a faster rate post-launch in the United States compared to Germany and Switzerland.^7,8^

It is important to understand the US biosimilar market and to what degree has biosimilar introduction lowered cost and increased patient choice. Originator product manufacturers may take different strategies in response to biosimilar introduction, with some offering larger pricing discounts than others to achieve sole preferred coverage of their product. Currently, it is unclear how these varying strategies influence overall cost savings and market shares.

In this study, we used four originator family case studies that best exemplified two coverage strategies to compare their impact—(1) sole preferred coverage (ie, aim is to have originator product preferred; biosimilar(s) non-preferred), defined by a decline in net prices by at least 50% following the introduction of biosimilar competition by 2022—infliximab and pegfilgrastim, and (2) non-sole preferred coverage (ie, aim is to have originator product preferred alongside biosimilar products), defined by a lack of net prices decline by at least 50% of its pre-biosimilar competition value—trastuzumab and filgrastim.

As expected, we found that in cases where originator manufacturers pursue non-sole preferred coverage strategies (as evident by a small reduction in originator product average sales price [ASP]), originator market shares declined, and payers facilitated biosimilar access via coverage policies. In contrast, in cases where originator manufacturers pursue sole preferred coverage strategies (indicated by a steeper decline in originator ASP), originator products maintained a majority market share within their family of products. Regardless of coverage strategy, we found that the overall ASP within a family of products (originator and biosimilars) declined following the introduction of biosimilars, suggesting that (1) current biosimilar policies are having the intended impact on reducing prices and (2) biosimilar utilization alone may not be a complete measure of a functioning competitive biologics market.

## Methods and data

### Data analysis and sources

We performed three analyses. First, we examined payer preference for each originator and corresponding biosimilars for each year from 2017 to 2022. For each drug and indication, we categorized each coverage policy as “preferred” if the given product was among the first-line options listed in the step therapy requirements, tracking the proportion of “preferred” options over time. The unit of analysis was a payer's policy for an originator or biosimilar product for a particular indication (eg, Aetna's policy for infliximab for ulcerative colitis).

Step therapy requirements come from the Tufts Medical Center Specialty Drug Evidence and Coverage (SPEC) Database, which includes specialty drug coverage policies from 18 large US commercial health plans (Appendix 2). This analysis tracks 17 of the 18 US commercial plans; Kaiser Permanente was added to the SPEC database in 2019 and was dropped from analysis as its coverage policies could not be tracked longitudinally. These health plans represent roughly 161 million lives, covering an estimated 70% of the US commercial payer market.

Second, we evaluated changes in originator product market share in each quarter following biosimilar introduction.

For market share for all originator product families, we used the real-world IQVIA Longitudinal Access and Adjudicated Dataset (LAAD) for the period of April 2015 through December 2022. The LAAD pharmacy dataset is a deidentified claims-level dataset sourced from several pharmacy points of sale serving approximately 300 million patients and represents an estimated 92% of prescriptions dispensed at retail pharmacies and approximately 70% of prescriptions through mail order pharmacies, depending on the specialty. The LAAD medical dataset is a deidentified claims dataset from various healthcare sources including clearinghouses/switch houses and practice management software, with >80% coverage for American Medical Association providers capturing physician in-office drug administration and hospital outpatient usage. The dataset does not capture hospital admission (inpatient) usage reimbursed via diagnosis-related groups. Inpatient services likely do not represent a large share of the market.

Third, we quantified changes in market share-weighted ASP to look at drug family pricing trends. We calculated market share-weighted prices for originator–biosimilar families by multiplying the price of a product each quarter by its market share within its respective family. Next, we calculated the percent change in price compared to the originator product price prior to the introduction of the first biosimilar. Finally, we averaged the percent change across all originator–biosimilar families to estimate change within the originator–biosimilar market.

For pricing data, we used the Center for Medicare and Medicaid Services (CMS) ASP Quarterly Payment files spanning from July 1 through September 30 for years 2017 through 2022. ASP reflects the market-based average price that a manufacturer sells their drug after any rebates and discounts, except for Medicaid and certain federal discounts and rebates.

### Independent variable of interest

While there is limited visibility into a manufacturer's coverage strategy, net pricing trends using metrics such as ASP—which includes manufacturer rebates, volume discounts, and other concessions—can provide insight into originator manufacturer's pursued payer coverage strategy. We selected four originator family case studies that best exemplified two payer coverage strategies pursued by manufacturers: (1) sole preferred coverage (ie, aim is to have originator product preferred; biosimilar(s) non-preferred), defined as steep ASP reductions for originator products (decline in net prices by at least 50% following the introduction of biosimilar competition by 2022)—infliximab and pegfilgrastim, and (2) non-sole preferred coverage (ie, aim is to have originator product preferred alongside biosimilar products), defined as moderate ASP reductions for originator products with (net prices did not decline by at least 50% of its pre-biosimilar competition value)—trastuzumab and filgrastim (Table 1).

**Table 1.** Originator products and coverage strategy categorization.

Brand (active ingredient)	First biosimilar launch date	Number of biosimilars available in 2022	Originator coverage strategy	Originator price change since first biosimilar launch by January 1, 2022	
				Net (%)	WAC (%)
Remicade (infliximab)	November 2016	3	Sole preferred	−54	+5
Neulasta (pegfilgrastim)	July 2018	4	Sole preferred	−54	+3
Herceptin (trastuzumab)	July 2019	5	Non-sole preferred	−19	0
Neupogen (filgrastim)	September 2015	2	Non-sole preferred	−4	+3

### Included therapies

We included four originator biologics and 15 corresponding biosimilars approved through the BPCIA of 2009 at the drug-indication level, excluding indications for which no biosimilars were granted Food and Drug Administration (FDA) approval (Appendix 1). For example, Neupogen is approved for acute radiation syndrome, while none of its biosimilars are. We therefore excluded Neupogen decisions for acute radiation syndrome from this analysis. For the included therapies, we identified 4948 total coverage policies from 2017 to 2022.

## Results

### Analysis 1—Payer preference for originator and biosimilars products over time, by originator family

#### Sole preferred coverage strategy

**Figure 1.**
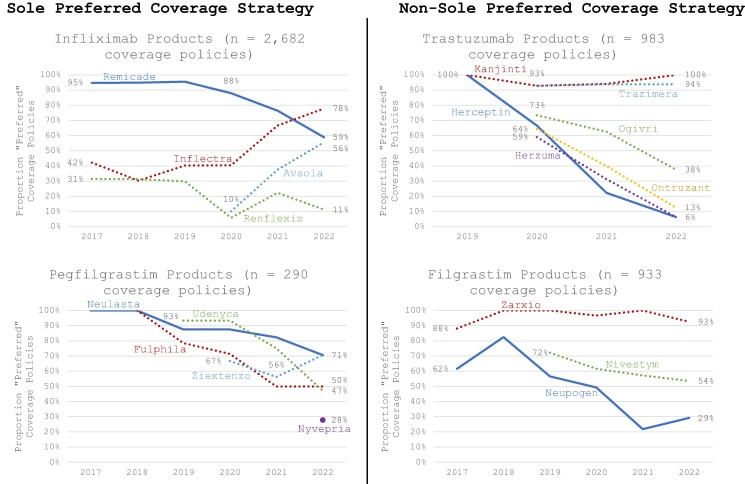
Payer preference for originator and biosimilar products, 2017-2022 (%). Source: Author's analysis of payer coverage data from the Specialty Drug Evidence and Coverage database.

Following the introduction of biosimilars, the proportion of payer decisions designating both Remicade (infliximab) and Neulasta (pegfilgrastim) as preferred declined (Figure 1, left panels, solid blue lines). Nonetheless, Remicade remained preferred more often than two of its three biosimilars (Figure 1, upper left panel), while Neulasta remained tied as most often preferred, compared to its biosimilars (Figure 1, lower left panel).

#### Non-sole preferred coverage strategy

Herceptin (trastuzumab) and Neupogen (filgrastim) saw steeper declines in payer preference following biosimilar introductions (Figure 1, right panels, solid blue lines). By 2022, both Herceptin and Neupogen were less often preferred relative to their biosimilars compared to Remicade and Neulasta (Figure 1).

### Analysis 2—Market share for originator and biosimilar products over time, by originator family

**Figure 2.**
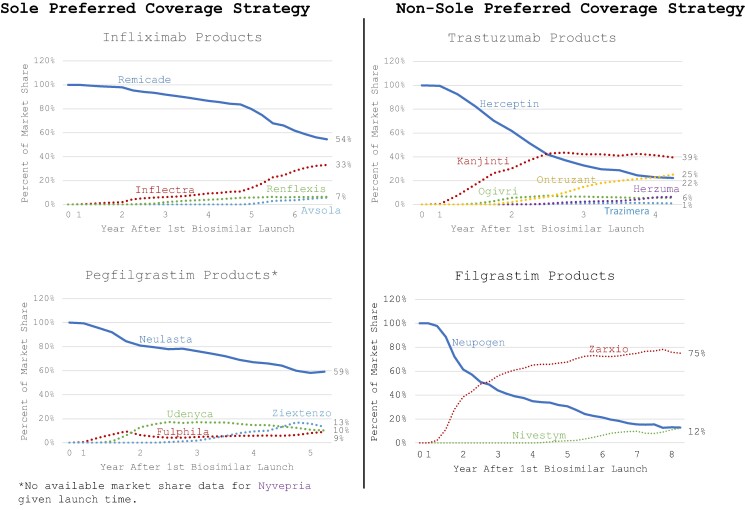
Market share for originator and biosimilar products, 2017-2022 (%). Source: Author's analysis of market share data from IQVIA Longitudinal Access and Adjudicated Dataset and PharMetrics Plus database (data on pharmacy and medical claims in each quarter from biosimilar entry).

Market shares of Remicade and Neulasta declined in the years following the release of their first biosimilar (Figure 2, left panels, solid blue lines). Remicade's market share decreased by 45% by the sixth year since the launch of its first biosimilar, Inflectra, while Neulasta's market share decreased by 40% by the fifth year since the launch of its first biosimilar, Fulphila. However, despite the availability of biosimilars, both originator products remained the dominant product within their respective family of drugs.

Herceptin’s and Neupogen's market shares declined substantially since the launch of their first biosimilars, Kanjinti and Zarxio, respectively (Figure 2, right panels, solid blue lines). At the end of the second year since launch, Kanjinti overtook the trastuzumab market and Zarxio overtook the filgrastim market. By the fourth year since trastuzumab biosimilar availability, market shares of Herceptin declined to 22% and by year eight of filgrastim biosimilar availability, market shares of Neupogen declined to 13%.

### Analysis 3—Pricing for originator and biosimilar products over time, by originator family

**Figure 3.**
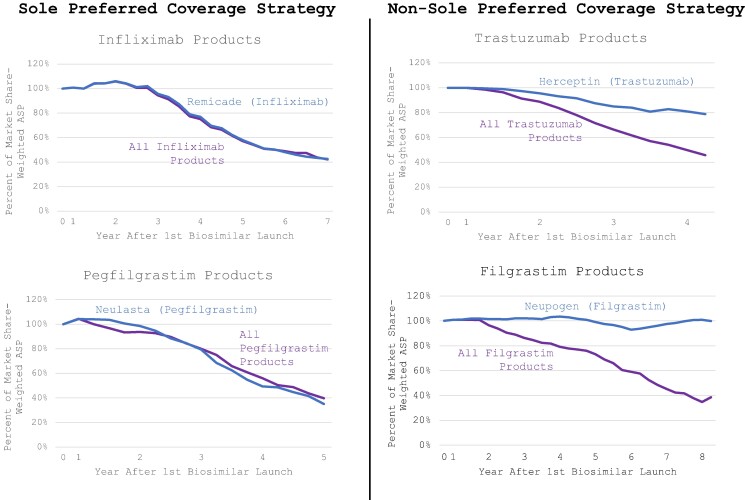
Weighted average sales price for originator and biosimilar products, 2017-2022 (%). Source: Author's analysis of weighted average sales price information from the Center for Medicare and Medicaid Services.

Consistent with the overall market (combined originator and biosimilars), market share-weighted ASPs declined significantly for originators with sole preferred coverage strategy, while originators pursuing non-sole preferred coverage strategies maintained higher market share-weighted ASPs. Regardless of coverage strategy, overall market share-weighted ASP for all included families significantly decreased in the years following the launch of their first biosimilar product (Figure 3).

## Discussion

Our research suggests that the availability of biosimilar products led to reduced ASP net prices of the product family (originator and biosimilars) regardless of whether the originator manufacturer has attempted to compete with their biosimilars for preferential coverage. Our analysis showed that originator products with sole preferred coverage strategies (largely through rebates) retained coverage as preferred products, while originators products with non-sole preferred coverage strategies did not. For example, in 2022, both Remicade and Neulasta were covered as preferred products within their respective family of drugs most of the time (59% and 71%, respectively), while some biosimilars for both Herceptin and Neupogen were preferred products within their family of drugs much less often (6% and 29%, respectively).

Originator products with sole preferred coverage strategies retained greater market share than those originator products with non-sole preferred coverage strategies. Conversely, biosimilar market share was higher in those families of drugs with non-sole preferred coverage strategies compared to those with sole preferred coverage strategies. Regardless of originator coverage strategy, the market-weighted ASP for all four product families (combined originator and biosimilars) declined by more than 50% in the years following the introduction of biosimilars competition, suggesting that biosimilar uptake may not be a complete measure of whether the biosimilar market is facilitating competition and lowering prices.

Our research indicates that payers have increasingly facilitated biosimilar access and that biosimilar market entry, and the resulting market competition, lowered ASP of both the originator and biosimilar products irrespective of biosimilar market share. These trends are consistent with a recent report from the Office of Inspector General (OIG)^9^ showing similar reductions in both originator and biosimilar prices post-biosimilar introduction in the Medicare Part B Program. These data add new considerations for what constitutes a functioning biosimilar market and challenge assumptions that cost savings are a result of increased biosimilar market share alone. Policy efforts, such as the Biosimilar Act of 2021, which authorizes the FDA to promote biosimilars and adequately equip patients and providers with tools to better utilize these products, are likely to further encourage biosimilar uptake.^10^ Continued monitoring of the biosimilar market remains important, as recently and soon-to-be introduced biosimilars (adalimumab and natalizumab) should significantly influence the market in their respective spaces. Further, the Inflation Reduction Act (IRA)^11^ of 2022 could influence incentives for future biosimilar development with an established ASP + 8% add-on payment rate for biosimilars under Medicare Part B. At the same time, it is unclear to what degree biologic negotiation with Medicare under the IRA will impact future biosimilar development.^12^

## Limitations

First, this work does not account for formulary tiering, which determines patient and plan cost-sharing and hence influences patient access. However, payers tend to cover the included biosimilars through their medical benefit, which is not subject to formulary tiering, so this limitation is unlikely to have had an important impact on our findings. The observed changes in ASP net price are likely largely driven by rebates or other concessions rather than declines in list price, and these savings are not always passed on to patients. It should be noted that CMS relies on manufacturer-provided data to calculate ASP and misreporting could affect accuracy. In a 2023 audit, the OIG found a small number of inconsistencies in manufacturers’ reporting.^13^ Second, we relied on average price data, which may differ from negotiated prices. Finally, we drew market share data from a broader set of payers than our coverage information of 17 commercial payers represents, which may not be generalizable to other payers and plan types.

## Conclusion

Trends in payer coverage, market share, and pricing reveal that biosimilar availability has yielded cost savings despite the adoption of different coverage strategies by originator product manufacturers. Furthermore, biosimilar uptake may not be a complete measure of whether the market is working as intended. Taken together, these findings indicate that the market for biologics is competitive and achieving cost savings. However, potential anti-trust behavior on the part of manufacturers warrants scrutiny, particularly in the use of exclusionary contracting with Pharmacy Benefit Managers (PBMs). By maintaining originator market shares through supply chain consolidation post-biosimilar launch, manufacturers, PBMs, and Group Purchasing Organizations could block biosimilar competition and limit their ability to generate cost savings.^14^ Future research should explore what impact originator price reductions, such as potentially with IRA, may have on the viability of investments into new biosimilar development. Policy efforts aimed at regulating prices should also consider the impact on the existing biosimilars market, which is vital to continue realizing the promise of lower costs and better access to biologic products in the United States.

## Supplementary Material

qxae090_Supplementary_Data
