# Supplementary material for: Biosimilar underutilization alone does not foretell a broken biologics market
Source: Health Aff Sch. 2024 Jul 17;2(7):qxae090. doi: 10.1093/haschl/qxae090 (PMC11282456; doi:10.1093/haschl/qxae090)
Supplement: qxae090_Supplementary_Data [file qxae090_supplementary_data.zip › Appendix.docx]

Appendix 1: Included Products

| **Brand** | **Active Ingredient** | **Launch Date** |
| --- | --- | --- |
| Remicade | Infliximab |  |
| Inflectra | infliximab-dyyb | 11/2016 |
| Renflexis | infliximab-abda | 07/2017 |
| Avsola | infliximab-axxq | 07/2020 |
| Neulasta | Pegfilgrastim |  |
| Fulphila | pegfilgrastim-jmdb | 07/2018 |
| Udenyca | pegfilgrastim-cbqv | 01/2019 |
| Ziextenzo | pegfilgrastim-bmez | 11/2019 |
| Nyvepria | pegfilgrastim-apgf | 01/2022 |
| Herceptin | Trastuzumab |  |
| Kanjinti | trastuzumab-anns | 07/2019 |
| Ogivri | trastuzumab-dkst | 12/2019 |
| Trazimera | trastuzumab-qyyp | 02/2020 |
| Herzuma | trastuzumab-pkrb | 03/2020 |
| Ontruzant | trastuzumab-dttb | 04/2020 |
| Neupogen* | Filgrastim |  |
| Zarxio | filgrastim-sndz | 09/2015 |
| Nivestym | filgrastim-aafi | 10/2018 |

* Granix (tbo-filgrastim) omitted given approval via traditional FDA biologic pathway

Appendix 2: US Commercial Health Plans Included in the SPEC Database

1. Aetna

2. Anthem

3. BCBS Florida

4. BCBS Massachusetts

5. BCBS Michigan

6. BCBS New Jersey

7. BCBS North Carolina

8. BCBS Tennessee

9. CareFirst BCBS

10. Centene

11. Cigna

12. Emblem Health

13. Health Care Service Corporation (HCSC)*

14. Highmark Health

15. Humana

16. Independence BC

17. Kaiser Permanente**

18. UnitedHealthcare

* HCSC is comprised of BCBS Plans in Illinois, Montana, New Mexico, Oklahoma, and Texas.

**Kaiser Permanente is listed under commercial plans in the SPEC database, but since it was added to the database in 2019 it will not be included in the longitudinal analysis.
